# TGFβ upregulates PAR-1 expression and signalling responses in A549 lung adenocarcinoma cells

**DOI:** 10.18632/oncotarget.11472

**Published:** 2016-08-22

**Authors:** Natalia Smoktunowicz, Manuela Platé, Alejandro Ortiz Stern, Vanessa D'Antongiovanni, Eifion Robinson, Vijay Chudasama, Stephen Caddick, Chris J. Scotton, Gabor Jarai, Rachel C. Chambers

**Affiliations:** ^1^ Centre for Inflammation and Tissue Repair, UCL Respiratory, University College London, London, UK; ^2^ Department of Chemistry, University College London, London, UK; ^3^ Novartis Institutes of Biomedical Research, Horsham, UK

**Keywords:** PAR-1, TGFβ, thrombin, cancer

## Abstract

The major high-affinity thrombin receptor, proteinase activated receptor-1 (PAR-1) is expressed at low levels by the normal epithelium but is upregulated in many types of cancer, including lung cancer. The thrombin-PAR-1 signalling axis contributes to the activation of latent TGFβ in response to tissue injury via an αvβ6 integrin-mediated mechanism. TGFβ is a pleiotropic cytokine that acts as a tumour suppressor in normal and dysplastic cells but switches into a tumour promoter in advanced tumours. In this study we demonstrate that TGFβ is a positive regulator of PAR-1 expression in A549 lung adenocarcinoma cells, which in turn increases the sensitivity of these cells to thrombin signalling. We further demonstrate that this effect is Smad3-, ERK1/2- and Sp1-dependent. We also show that TGFβ-mediated PAR-1 upregulation is accompanied by increased expression of integrin αv and β6 subunits. Finally, TGFβ pre-stimulation promotes increased migratory potential of A549 to thrombin. These data have important implications for our understanding of the interplay between coagulation and TGFβ signalling responses in lung cancer.

## INTRODUCTION

Lung cancer is a global health problem with 1.5 million people being diagnosed worldwide every year. This condition accounts for 18% of all deaths from cancer, which makes it the leading cause of cancer mortality [1]. Cancer patients have a sevenfold increased risk of venous thromboembolism [2] and cancer-related coagulopathy is associated with advanced malignancy and correlates positively with increased mortality rates [3]. Cancer cells constitutively express tissue factor [4], the main initiator of the extrinsic coagulation pathway, and the leaky nature of the tumour vasculature contributes to the accumulation of thrombin in the tumour microenvironment [5]. Thrombin exerts a plethora of cellular effects via the activation of proteinase activated receptor-1 (PAR-1). PAR-1 belongs to a family of four G-protein coupled receptors (PAR 1-4) that are activated via a unique mechanism involving proteolytic cleavage of the N-terminus. This results in the unmasking of a tethered ligand, which then binds intramolecularly to the receptor and initiates cell signalling via the phosphorylation of the associated G-protein complex [6]. PAR-1 forms stable complexes with Gα_i/o_, Gα_q/11_, and Gα_12/13_ and thereby links to multiple second messenger pathways and influences multiple cellular responses [7].

PAR-1 is expressed at low levels by the normal epithelium but is upregulated in many types of cancer, including lung [5], gastric [8], ovarian [9], prostate [10], melanoma [11], breast [12] and colon cancer [13]. PAR-1 and vascular endothelial growth factor (VEGF) overexpression in lung cancer has been associated with increased metastatic potential [14] and reduced three-year survival in non-small cell lung cancer [15]. PAR-1 signalling also regulates the expression and leads to the activation of other receptors that contribute to cancer progression. These include platelet-activating factor receptor (PAFR) that causes platelet mobilisation and promotes tumour metastases [16], as well as epidermal growth factor receptor (EGFR) which also promotes tumour invasion [17].

PAR-1 has been strongly linked to the integrin mediated activation of latent TGFβ, a pleiotropic cytokine that is a tumour suppressor in normal and dysplastic cells but turns into a tumour promoter in advanced cancer cells [18]. This mode of activation is highly cell-type specific and is mediated via an αvβ6 integrin-dependent mechanism in epithelial cells [19, 20]; whereas in fibroblasts, this mode of activation is mediated via an αvβ5 integrin-dependent mechanism [21]. Both modes of activation are dependent on the mechanotransduction of cytoskeletal tension within the cell, which in concert with matrix interactions leads to a change in the conformation of the latent TGFβ complex and release of active TGFβ [19, 21–23].

Human tumours overproduce TGFβ, which can lead to the loss of epithelial markers, such as E-cadherin, and epithelial-to-mesenchymal transition (EMT), an event which has been linked to increased tumour cell survival, motility and invasiveness [24]. This is also associated with modulation of tumour cell-stroma interactions through regulation of integrin expression [25], increased production of the matricellular protein, connective tissue growth factor (CTGF) [26] and re-modelling of the extracellular matrix [27]. The αvβ6 integrin is upregulated in many cancer cells and targeting αvβ6 leads to reduced TGFβ activation and cancer cell proliferation [28].

The importance of PAR-1 in mediating TGFβ activation *in vivo* is gaining increasing recognition [19, 21] but the effect of TGFβ signalling on PAR-1 expression remains largely unexplored. In this study we investigated the interaction between TGFβ signalling and PAR-1 expression and functional activity in A549 lung adenocarcinoma cells. We show for the first time that TGFβ increases PAR-1 gene, protein and cell surface expression and that this in turn results in increased A549 cell responsiveness to subsequent thrombin stimulation. These findings shed important light on the interplay between coagulation and TGFβ signalling responses and further provide a potential novel mechanistic model by which these pathways may interact to promote lung cancer progression.

## RESULTS

### TGFβ increases PAR-1 expression and renders A549 cells more responsive to thrombin stimulation

**Figure 1:**
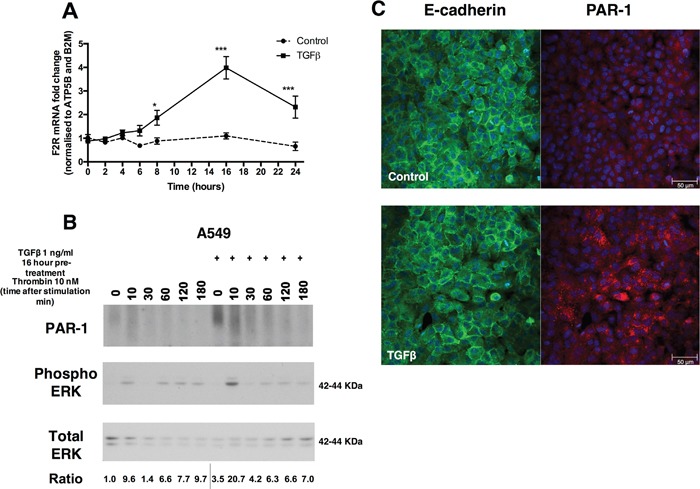
TGFβ induces the expression of PAR-1 in A549 lung adenocarcinoma cells **Panel A.** Human lung adenocarcinoma A549 cells were exposed to TGFβ (1 ng/ml) and mRNA collected at indicated times over the course of 24 hours. PAR-1 expression was quantified by real-time qPCR. Each data point represents the mean +/− SEM of 3 replicate wells, statistically analysed by Two-way ANOVA, **p<0.01, ***p<0.001 in comparison to control. **Panel B.** Western blot analysis of PAR-1 protein expression and phosphorylated and total ERK 1/2 levels following exposure to TGFβ (1 ng/ml) for 16 hours and subsequent thrombin (10 nM) stimulation at indicated times. Representative immunoblots with quantitated ratios phosphorylated ERK/total ERK. The PAR-1 predicted molecular weight is 47 kDa but it is detected in most mammalian cells as a broad high molecular weight species due to differential N-linked glycosylation (63). **Panel C.** Immunocytofluorescence visualisation of E-cadherin (green) and PAR-1 (red) following exposure to TGFβ (1 ng/ml) for 24 hours. DAPI was used to visualise the nuclei. The top panel shows images for control cells and lower panel show the staining of TGFβ-stimulated cells, x20 original magnification.

A549 cells express low levels of PAR-1 under baseline conditions. Exposure to TGFβ (1 ng/ml) leads to a time-dependent upregulation of *F2R/*PAR-1 mRNA levels from 8 hours onwards with a 4-fold maximal increase at 16 hours (Figure 1A). By 16 hours of TGFβ exposure, PAR-1 protein levels are also increased and subsequent thrombin-mediated PAR-1 stimulation led to increased ERK phosphorylation at 10 minutes when compared to control cells (Figure 1B). This increase in PAR-1 protein levels occurs with no evidence of E-cadherin loss as determined by immunocytofluorescence at 24 hours (Figure 1C). These data suggest that the increase in PAR-1 expression in response to TGFβ stimulation for 24 hours is not associated with EMT.

**Figure 2:**
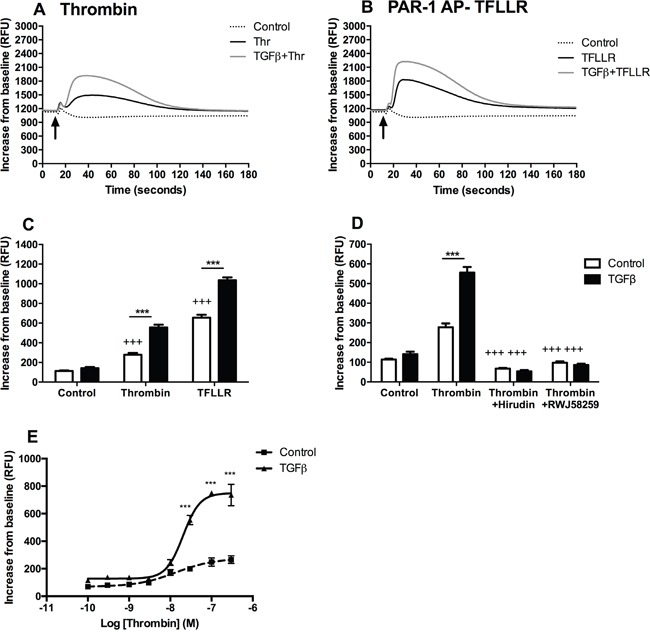
TGFβ-mediated PAR-1 upregulation enhances A549 lung adenocarcinoma cell responsiveness to thrombin signalling Intracellular calcium release was monitored in A549 cells, pre-treated with TGFβ (1 ng/ml), in response to thrombin (30 nM) and PAR-1 activating peptide, TFLLR (P1 AP, 100 μM). **Panel A.** Representative traces for thrombin-mediated calcium flux; **Panel B.** P1 AP TFLLR. **Panel C.** Analysis of intracellular calcium responses mediated by PAR-1 agonists. **Panel D.** Thrombin signalling was inhibited directly by pre-incubation with hirudin (150 nM) and by using a PAR-1 antagonist, RWJ58259 (3 μM) and calcium flux was analysed. **Panel E.** Concentration-response curve to thrombin in TGFβ-pre-treated (solid line) and control (dotted line) A549 lung adenocarcinoma cells. Each data point represents the mean +/− SEM of 3-4 replicate wells from at least two independent experiments. Two-way ANOVA was performed for statistical analysis, ***p<0.001 comparison between control and TGFβ-pre-treated cells; +++p<0.001 comparison between treatments.

Consistent with low protein expression of PAR-1 in A549 cells at baseline, stimulating cells with thrombin and the PAR-1 activating peptide, TFLLR, produced a delayed and modest release of intracellular calcium as determined by monitoring changes in fluorescence following incubation with Fluo4-AM (Figure 2A and 2B). Pre-stimulation with TGFβ significantly increased subsequent calcium responses to either thrombin or TFLLR (Figure 2C). These effects were inhibited when thrombin was rendered catalytically inert with hirudin, as well as in the presence of the small molecule PAR-1 antagonist, RWJ58259 (Figure 2D). Moreover, pre-treatment with TGFβ increased the functional responsiveness of A549 in an agonist concentration-dependent manner (Figure 2E). Under baseline conditions, A549 cells mount a small, albeit significant, increase in intracellular calcium release with increasing concentrations of thrombin. In contrast, pre-exposure to TGFβ resulted in a 4-fold increase in the magnitude of the calcium response. Taken together these data led us to conclude that TGFβ increases PAR-1 expression and the subsequent magnitude of the calcium response to thrombin.

### TGFβ regulation of PAR-1 expression is Smad3-, MEK- and SP1-dependent

**Figure 3:**
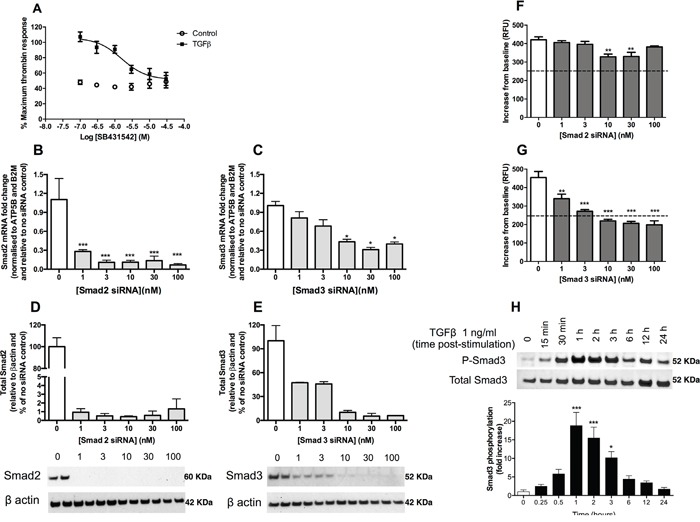
TGFβ upregulates PAR-1 expression in A549 lung adenocarcinoma cells via an ALK5-Smad3-dependent mechanism **Panel A.** Dose-inhibition curve in A549 cells exposed to TGFβ (1 ng/ml) with varying concentrations of TGFβ receptor (ALK5) inhibitor, SB431542, for 24 hours and then stimulated with thrombin. **Panels B** and **C.** Smad2 and Smad3 siRNA knockdown measured by mRNA (real-time qPCR, each data point represents the mean +/− SEM of 3 replicate wells; one-way ANOVA, *p<0.05, ***p<0.001, relative to non-transfected control) and **Panels D** and **E.** Smad2 and Smad3 protein knockdown 48 hours post-transfection (Western blot, semi-quantitative densitometry analysis of the total protein of interest relative to the β-actin loading control, each data point represents the mean +/− SEM of 2 replicates as shown on the blots). **Panels F** and **G.** Intracellular calcium release was measured in A549 cells treated with varying concentrations of Smad2 and Smad3 siRNA, respectively, for 48 hours prior to stimulation with TGFβ (1 ng/ml) for 24 hours. Cells were incubated with Fluo-4AM calcium binding dye for one hour before being exposed to thrombin (30 nM). Each data point represents the mean +/− SEM of 3-4 replicate wells; Two-way ANOVA, ***p<0.001 comparison of control and TGFβ pre-treated cells. **Panel H.** A549 cells were incubated with or without TGFβ (1 ng/ml) for up to 24 hours. Representative immunoblots of total and phosphorylated Smad3 and quantification of relative intensity of pSmad3 immunoreactive bands by densitometry. Data shown are mean ± SEM of 3 independent experiments and expressed as fold increase over control cells, analysed by one-way ANOVA, *p<0.05, ***p<0.001.

We next examined the potential mechanism by which TGFβ mediates these effects in A549 cells. TGFβ signals via the formation of a heterotetrameric receptor complex composed of a TGFβ type 2 receptor dimer with an activin-like kinase (ALK) TGFβ type 1 receptor dimer; this in turn triggers the Smad signalling pathway by phosphorylating Smad2 and Smad3. In the current study, we show that the effect of TGFβ pre-treatment on subsequent thrombin-mediated calcium release in A549 cells is inhibited in a concentration-dependent manner in the presence of the ALK5 receptor inhibitor, SB431542, (IC_50_ = 1.7 μM) (Figure 3A).

The role of Smad2 and Smad3 in TGFβ-mediated PAR-1 upregulation was subsequently investigated using small interfering RNA (siRNA). The silencing of Smad2 and Smad3 expression was validated at the mRNA (Figure 3B and 3C) and protein levels (Figure 3D and 3E). Although Smad2 knockdown was found to exert a small but significant inhibitory effect on the subsequent TGFβ-mediated increase in PAR-1-mediated intracellular calcium release at 48 hours at siRNA concentrations of 10 nM and 30 nM, the intracellular calcium flux remained significantly elevated above the unstimulated control cells exposed to thrombin (Figure 3F). In contrast, silencing of Smad3 expression significantly reduced the TGFβ-mediated increase in subsequent PAR-1-mediated signalling responses to thrombin in a concentration-dependent manner (Figure 3G). Examination of the kinetics of Smad3 phosphorylation in A549 cells revealed that Smad3 phosphorylation increases from 30 minutes following TGFβ exposure onwards, peaking at 1 hour with a near 20-fold increase (p<0.001 versus control), before returning to control levels by 24 hours (Figure 3H). Taken together these data led us to conclude that TGFβ mediates the increase in PAR-1 expression and functional responses via an ALK5/Smad3-dependent mechanism.

**Figure 4:**
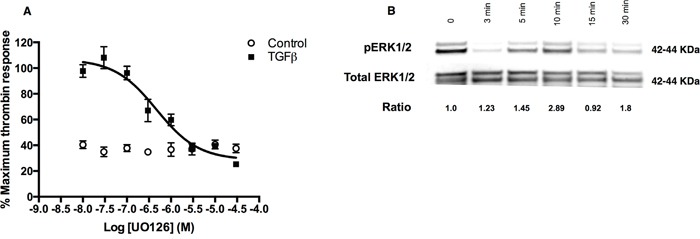
TGFβ-ERK1/2 signalling is also involved in regulation of PAR-1 expression in A549 cells **Panel A.** Dose-inhibition curve in A549 cells exposed to TGFβ (1 ng/ml) with varying concentrations of MEK1/2 inhibitor, UO126, for 24 hours and then stimulated with thrombin (30 nM). Each data point represents the mean +/− SEM of 3-4 replicate wells. **Panel B.** Representative immunoblot of ERK1/2 phosphorylation in A549 cells incubated with TGFβ (1 ng/ml) for up to 30 minutes with corresponding pERK/total ERK ratios derived from densitometry analysis.

TGFβ signalling responses are often dependent on cross-talk with Smad-independent pathways, including ERK, SAPK/JNK, and p38 MAPK pathways. Our studies revealed that the MEK1/MEK2 inhibitor, UO126, inhibited the TGFβ-mediated increase in thrombin-stimulated intracellular calcium flux in a concentration-dependent manner with an IC_50_ of 0.35 μM (Figure 4A). In contrast, inhibition of JNK, p38 and Rho kinase did not affect TGFβ-mediated increase in PAR-1 expression and signalling (data not shown). We also confirmed that TGFβ signalling in A549 cells leads to ERK1/2 phosphorylation, which peaks early at 10 minutes post-stimulation (Figure 4B).

**Figure 5:**
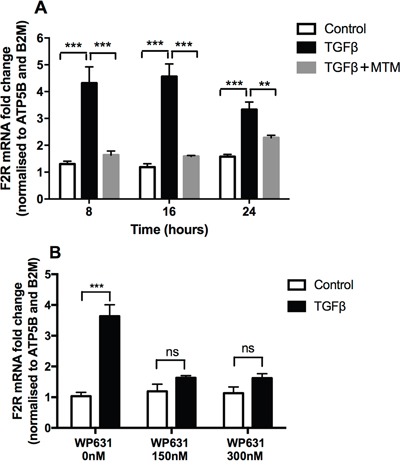
TGFβ-mediated PAR-1 upregulation is blocked by Sp1 inhibitors A549 lung adenocarcinoma cells were incubated with or without TGFβ (1 ng/ml) for 24 hours and in the presence of the Sp1 inhibitors. **Panel A.** Mithramycin A (10 μM) for 8, 16 and 24 hours, **Panel B.** WP631 for 16 hours at the concentration 150 nM and 300 nM. PAR-1 expression was quantified by real-time qPCR. Each data point represents the mean +/− SEM of 3 replicate wells, analysed by Two-way ANOVA, **p<0.01, ***p<0.001 in comparison to vehicle control.

We next addressed the question of which transcription factor might be involved downstream of Smad3 and ERK signalling. We focused our studies on the potential role of specificity protein 1 (Sp1) since this transcription factor has been linked to increased *F2R* promoter region binding [11], is known to interact with Smad3 [29] and is also implicated in carcinogenesis [30]. Our studies revealed that mithramycin A and WP631, two inhibitors that specifically displace Sp1 from DNA, were highly effective at blocking the TGFβ-induced increase in PAR-1 mRNA levels (Figure 5A and 5B).

### TGFβ increases integrin expression in A549

**Figure 6:**
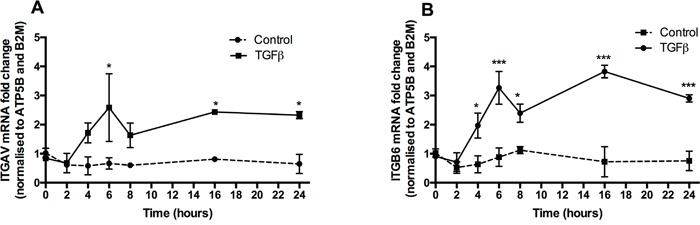
TGFβ promotes αv and β6 integrin sub-unit gene expression **Panels A** and **B.** A549 lung adenocarcinoma cells were incubated with or without TGFβ (1 ng/ml) for 24 hours. The mRNA was collected at indicated times in the course of 24 hours. Integrin subunits αv and β6 expression was quantified by real-time qPCR. Each data point represents the mean +/− SEM of 3 replicate wells, analysed by Two-way ANOVA, **p<0.01, ***p<0.001 in comparison to control.

We next examined the potential functional consequences of TGFβ-induced PAR-1 expression. PAR-1 activation has been strongly linked to the integrin-mediated activation of TGFβ via the αvβ6 integrin in epithelial cells [19] and the αvβ5 integrin in fibroblasts [21]. Examination of these integrin subunit mRNA levels in A549 cells following stimulation with TGFβ revealed that the αv and β6 subunits were significantly upregulated from 6 and 4 hours onwards, respectively (Figure 6A and 6B) and that both integrin subunits remained significantly elevated throughout the duration of the experiment (24 hours). Taken together these data demonstrate that TGFβ-induced upregulation of PAR-1 expression is accompanied by increased expression of the major integrin subunits involved in the activation of the latent form of this cytokine.

### TGFβ increases A549 migratory potential via PAR-1

**Figure 7:**
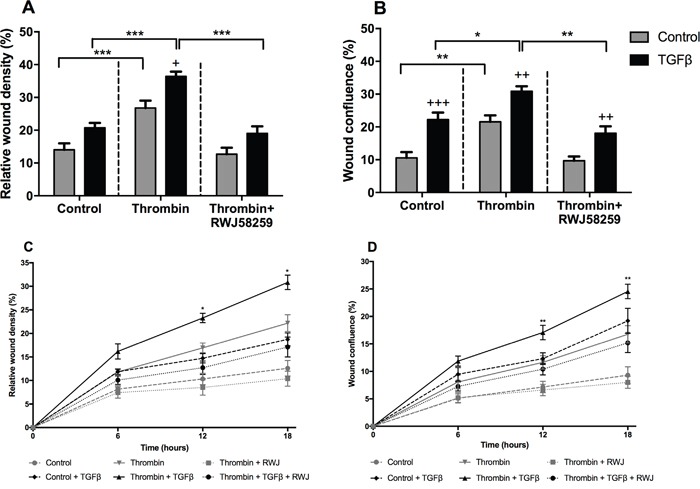
The effect of TGFβ treatment on A549 lung adenocarcinoma cells migration A549 cells were incubated with or without TGFβ (1 ng/ml) for 24 hours and pre-treated with PAR-1 inhibitor, RWJ58259 (3 μM) for 30 minutes. Homogenous scratch wounds were introduced using WoundMaker (Essen Bioscience, UK) and cells immediately stimulated with thrombin (10 nM). Wound closure was monitored for up to 24 hours and data analysed using Incucyte Zoom Live Cell Imaging System (Essen Bioscience, UK). **Panel A.** Difference in wound cell density and **Panel B.** Wound confluence at 24 hours. **Panel C** and **D.** Time-course of wound closure with data collected at 6, 12 and 18 hours with control and control + TGFβ denoted by dashed lines, thrombin and thrombin + TGFβ denoted by continuous lines and thrombin + RWJ58259 and thrombin + TGFβ + RWJ58259 denoted by dotted lines. Data are plotted as mean +/− SEM of n=5 replicate wells from 2 independent experiments; Two-way ANOVA, +p<0.05, ++p<0.01, +++p<0.001 in comparison to control, *p<0.05, **p<0.01, ***p<0.001 comparison between stimulations.

We further examined A549 cell motility in response to PAR-1 activation following TGFβ pre-treatment. Identical scratch wounds were introduced in confluent A549 cell monolayers. Cell migration was monitored over 24 hours and reported as wound confluence and cell density (Figure 7A and 7B). We observed that TGFβ and thrombin independently increased the rate of cell migration in A549 cells when compared with untreated cells at 24 hours. Subsequently, cells exposed to TGFβ and then stimulated with thrombin showed the highest rate of migration in this model. Inhibition of PAR-1 signalling with RWJ58259 abrogated this response. We further investigated the dynamics of this response by investigating the time-course of A549 migration in the wound assay (Figure 7C and 7D). Data collected at 6, 12 and 18 hours confirmed that cells exposed to TGFβ have a faster migration rate compared with control stimulated cells throughout the experiment. Furthermore, RWJ58259 inhibited the thrombin-mediated migration at 12 and 18-hour time points (p<0.01) while the TGFβ-thrombin-RWJ58259 treated cells migrated at a rate which was similar to TGFβ alone stimulated cells. These data led us to conclude that the additive effect of these two independent pathways increases A549 motility.

## DISCUSSION

The PAR receptors act as sensors of extracellular proteinase gradients and allow cells to react and respond to proteolytic changes within their microenvironments [12]. PAR-1, the prototypic member of the PAR family, has been identified as an oncogene and has been reported to promote cancer invasion and metastasis in the context of multiple cancers, including breast, colon, lung, pancreatic and prostate cancer as well as melanoma [14, 31–35]. Furthermore, PAR-1 expression levels positively correlate with the degree of cancer invasiveness [33]. TGFβ signalling similarly regulates a plethora of cell responses, including cell growth, differentiation, apoptosis, motility, invasion, ECM production, angiogenesis, and immune responses [36]. In the setting of cancer, TGFβ plays a dichotomous role in that it acts as a tumour suppressor in early tumorigenesis and as an oncogene in the later phases of tumour progression [37].

The aim of the present study was to explore the interplay between PAR-1 and TGFβ signalling responses in the context of lung cancer. Using A549 adenocarcinoma cells, we show for the first time that TGFβ increases the expression of PAR-1 and thereby leads to increased cellular responsiveness to thrombin. Thrombin is regarded as a key mediator in cancer growth and metastatic spread, via both the formation of fibrin and via its plethora of PAR-1 mediated cellular responses, including effects on DNA synthesis and cell proliferation, platelet activation, vascular permeability, cell migration, and induction of pro-angiogenic and pro-metastatic factors, such as VEGF (Reviewed in [38]).

The TGFβ receptors are constitutively expressed and form heterotetrameric TβRII-ALK5 ligand-receptor complexes upon ligand binding. This leads to phosphorylation of specific serine and threonine residues and activation of canonical TGFβ signalling via the phosphorylation of Smad2 and Smad3 that, upon binding to co-factor Smad4, translocate to the nucleus and influence target gene expression. In the present study, using ALK5 small molecule inhibition and siRNA knockdown of Smad2 and Smad3, we demonstrate that the upregulation of PAR-1 expression is both ALK-5 and Smad3-dependent in A549 adenocarcinoma cells. Interestingly, the TGFβ-Smad3 signalling pathway has previously been shown to promote cancer cell survival and metastasis [39].

TGFβ bound to TβRII-ALK5 complexes also leads to phosphorylation of tyrosine residues and the recruitment of adaptor molecules involved in the activation of MAPK kinase (ERK, JNK and p38) signalling [40]. TGFβ-dependent ERK signalling has been linked to EMT in cancer cells [41] and genetic profiling of TGFβ-mediated EMT revealed that ERK participates in the regulation of the expression of cell-matrix adhesion and motility genes [42]. In the present study, we show that the TGFβ-mediated increase in PAR-1 functional responses (calcium signalling) is also MEK-dependent; with no role identified for JNK or p38 signalling. It has previously been reported that at least 48 hours of prolonged exposure to TGFβ is necessary for a complete loss of epithelial marker expression and evidence of mesenchymal marker expression in cancer cells [43]. PAR-1 expression is low in lung epithelial cells; whereas fibroblasts express PAR-1 abundantly [44, 45]. In our study we note that the increase in PAR-1 expression in A549 cells occurs within 24 hours of TGFβ stimulation when E-cadherin is still highly expressed, indicating that acquisition of PAR-1 expression precedes the EMT process. Interestingly, gastric cancer cells [46] and A549 cells [47] have been shown to undergo thrombin-induced EMT via PAR-1 and ERK 1/2 activation, albeit over longer period of time (72 hours). In our study, we show that increased PAR-1 expression following exposure to TGFβ is also associated with increased ERK phosphorylation in response to thrombin stimulation. Taken together our findings combined with existing data in the literature point to the possibility that the early TGFβ-mediated increase in PAR-1 expression and subsequent increased responsiveness to thrombin could be a part of the EMT process.

In terms of the transcriptional regulation of PAR-1 expression, the transcription factors involved remain to be fully characterized but appear to be highly cell-specific. Moreover, PAR-1 expression is differently regulated during homeostasis and in disease, and displays both cell- and tissue-specific variations. The *F2R* gene promoter has been characterised and presents, among many transcription factor binding elements, two AP-2-Sp1 complexes at its proximal 3′ end. Overall, these complexes contain 3 AP-2 binding sites and 7 Sp1 binding sites [48]. In human melanoma cells, AP-2 has been shown to repress PAR-1 expression and compete with the positive regulator Sp1. The loss of AP-2 and predominant binding of Sp1 is linked to the overexpression of PAR-1 and the metastatic phenotype of these cells [49]. This evidence together with the reported observation that Smad3 can form transcriptional complexes with Sp1 to regulate gene expression [50], led us to focus our investigation on Sp1 as a candidate transcription factor in mediating TGFβ-induced PAR-1 upregulation in A549 adenocarcinoma cells. These studies revealed that the widely used Sp1 inhibitor, mithramycin A, that binds to GC rich DNA sequences [51–56], completely blocked the TGFβ induced increase in PAR-1 mRNA levels. We further confirmed the role of Sp1 in TGFβ-mediated PAR-1 regulation by using a second Sp1 inhibitor, the bisintercalating anthracycline, WP631, which has high specificity and binding affinity for Sp1-DNA binding sites at concentrations within the nanomolar range [57, 58].

We next sought to determine whether TGFβ-induced PAR-1 expression is accompanied by an increase in αvβ6 integrin expression. This integrin is expressed at low levels in normal epithelium but is known to be upregulated in response to tissue injury [19] and cancer [59]. Moreover, blocking ανβ6 integrin and TGFβ signalling has been shown to reduce tumour invasiveness [28]. In the present study we found that the increase in PAR-1 expression following TGFβ stimulation in A549 cells is accompanied by a concomitant upregulation of αv and β6 integrin subunit expression. It is important to note that the upregulation of the expression of these three genes did not require PAR-1 activation so that the upregulation of integrin expression is TGFβ dependent but PAR-1-independent. It is tempting to speculate that the interplay of this TGFβ-PAR-1 axis might potentially lead to a feed-forward mechanism, which could drive the perpetuation of ανβ6-mediated TGFβ activation and signalling in the presence of uncontrolled coagulation.

Finally, in order to further investigate the functional consequences of increased PAR-1 expression in response to TGFβ exposure in A549 cells, we performed a cell motility assay and show that there was a significant, additive effect on cell migration when TGFβ–pre-treated cells were stimulated by thrombin. These additive effects were abolished in the presence of the PAR-1 inhibitor. Although there is existing evidence that PAR-1 and TGFβ independently promote cancer cell motility and migration [12, 18, 60], to the best of our knowledge, this represents the first report of a potential novel mechanism by which the TGFβ and PAR-1 signalling pathways may converge to promote cancer cell function.

### Conclusion and therapeutic implications

In conclusion, our data show for the first time that TGFβ is a potent inducer of PAR-1 expression in lung adenocarcinoma cells. These effects are mediated via canonical TGFβ signalling with ALK5 and Smad3 acting in cooperation with an ERK-dependent mechanism and Sp1-dependent gene transcription. The upregulation of PAR-1 is accompanied by increased αv and β6 integrin subunit expression providing a potential mechanism for prolonged TGFβ activation in the pro-coagulant environment. These findings provide a scenario for the convergence of TGFβ and coagulation signalling to promote cancer cell function and migration. Future studies with an orally available, potent, PAR-1 antagonist (SCH530348/vorapaxar; trade name, Zontivity) which was recently approved by the U.S. Food and Drug Administration to reduce the risk of heart attacks and stroke in high-risk patients [61], as well as αvβ6 integrin blocking agents, which are currently in clinical development [62], may offer therapeutic approaches for lung adenocarcinoma and potentially other cancers.

## MATERIALS AND METHODS

### Reagents and inhibitors

Commercially-available A549 cells were characterised and sourced from ATCC (LGC Standards, UK). Human α-thrombin was purchased from Enzyme Research Laboratories, UK. Transforming growth factor β-1 (TGFβ-1) was purchased from R&D Biosystems, UK. PAR-1 agonist peptide TFLLR-NH_2_ was purchased from Bachem AG, Switzerland. Hirudin was purchased from Sigma, UK. MEK inhibitor, UO126, and ALK5 inhibitor, SB431542 were purchased from Calbiochem, UK. PAR-1 ATAP2 detection antibody was purchased from Santa Cruz Biotechnology, USA. Total Smad2 and Smad3, and phosphorylated Smad2 and Smad3, ERK and phosphorylation ERK antibodies were purchased from Cell Signalling Technologies, USA. PAR-1 inhibitor, RWJ58259, was synthesised in-house by the Department of Chemistry, UCL. Mithramycin A was purchased from VWR International, UK and WP631 was purchased from Insight Biotechnology, UK.

### RT-PCR and real-time RT-PCR analysis

Total RNA from cell culture lysates was isolated with TRIzol reagent as per manufacturer's protocol (Invitrogen, UK). Contaminating genomic DNA was removed using the Ambion DNAfree kit and cDNA was prepared by reverse-transcription (RT) using the qScript cDNA SuperMix® kit (Quanta Biosciences, USA) following the manufacturer's instructions. Real time RT-PCR was conducted using the Platinum SYBR Green qPCR SuperMix UDG (Invitrogen, UK) with ~1 ng of cDNA, and in-house designed forward and reverse primers (Table 1) each at a final concentration of 800 nM, on a Mastercycler EP Realplex (Eppendorf, Germany) at primer annealing temperature of 60°C. The GeNorm algorithm was applied to produce the optimal normalisation factor and two housekeeping genes, ATP synthase, H^+^ transporting, mitochondrial F1 complex, beta polypeptide (ATP5B) and beta-2-microglobulin (B2M), showed the highest reference target stability for human cells. Housekeeping primer, Smad2 and Smad3 primer mixes were purchased from PrimerDesign (Southampton, UK). To examine the quantitative differences in target mRNA expression in each sample, Cp values were determined from the linear region of the logarithmic amplification plot. The fold change was subsequently calculated using the standard 2^−ΔΔCp^ approach. Statistical analysis was performed using the ΔCp values.

**Table 1:** Primers

Gene	Forward sequence	Reverse sequence
**hPAR-1**	AGGCCAGAATCAAAAGCAAC	TCATCCTCCCAAAATGGTTC
**hITGAV**	TCTGTGCCGCGCCTTCAACC	AACATCCGGGAAGACGCGCTG
**hITGB6**	AAGTTGAGACCAGGTGGTGCGC	CCATGGAGGCGGAGAGGTCCAT

### Transfection with siRNA

SMARTpool ONTARGETplus Human siRNA targeting Smad2 and Smad3 and the ONTARGETplus Nontargeting Pool (Thermo Scientific, UK) were reconstituted to 20 μM stock in RNA buffer. A range of siRNA concentrations (100 nM, 30 nM, 10 nM, 3 nM, 1 nM) was tested to optimise the conditions and combined with Interferin transfection reagent (Polypus, UK) as per the manufacturer's protocol. Following reverse transfection protocol, A549 cells were combined with the transfection mix and immediately seeded. Cells were then incubated for 48 hours, after which the medium was removed and the samples collected for mRNA and protein analysis or replaced with serum-free medium for another 24 hours before cells were stimulated.

### Western blot

Cells were seeded in 24-well plates and following appropriate treatment, cells were washed with cold PBS and lysed in 100 μl of phosphosafe extraction buffer (Merck Chemicals, UK) supplemented with a complete protease inhibitor cocktail. BCA assay was used to measure protein concentration as per the manufacturer's protocol and the sample mixed with Laemmli buffer containing DTT. Following protein denaturation, the samples were loaded onto a pre-cast 4-12% polyacrylamide gel (Bolt Bis-Tris Plus gels, Novex, Life Technologies, UK). A Plus Prestained Protein Ladder (Fermentas, UK) with 10-250 kDa detection range was run in a separate lane to identify the molecular weights of individual proteins. Electrophoresis was performed at 165 V for 35 minutes. Dry protein transfer onto polyvinyldene difluoride (PVDF) membrane was performed using the iBlot Gel Transfer Device (Novex, Life Technologies, UK) at 20 V for 7 minutes. The membrane was subsequently incubated in blocking buffer, followed by primary detection antibodies for the protein of interest and finally with a specific horseradish peroxidise (HRP)-linked secondary antibody. The membrane was developed by enhanced chemiluminescence (ECL) following the manufacturer's instructions (GE Healthcare, UK). Immunoreactive protein bands were visualised by exposing the membrane to autoradiography film developer (Kodak, UK) and the exposure time adjusted to the strength of the signal.

### Intracellular calcium measurement

Cells were seeded 48 hours prior to the experiment in black 96-well plates with a clear bottom. On the day of the assay 1 vial of Fluo 4-AM dye was resuspended in 10 ml of HBSS/Hepes assay buffer without calcium or magnesium (Invitrogen, UK). Cells were incubated with the dye, 100 μl/well, for 30 minutes at 37°C and calibrated for another 30 minutes at room temperature. The PAR-1 antagonists used in this study were added and incubated with the dye for an hour prior to the experiment. PAR-1 agonists were prepared at 3x final desired concentration in assay buffer and 50 μl per well of appropriate compound was added to a 96-well agonist plate. Changes in the intracellular Ca^2+^ were monitored using a fluorescent image plate reader FLIPR Tetra (Molecular Devices, USA) and the results displayed and analysed using the ScreenWorks software. The device is fitted with a standard ECCM camera that records the fluorescent signal produced by light-emitting diodes (LED) that excites the Fluo-4AM dye at wavelength of 494 nm with emission at 516 nm. The fluorescence was monitored for 10 seconds before the agonists were simultaneously dispensed to the 96-well plate by the overhead pipettor. Readings were recorded every second for the first 60 seconds and every 6 seconds for the next 120 seconds. The change in intracellular calcium [Ca^2+^] was quantified as an increase in relative fluorescence units (RFUs). The difference between maximum and minimum RFU was used to plot the results.

### Immunocytofluorescence

Cells were seeded in 8-well chamber slides (Fisher Scientific, UK) and cultured for 48 hours in a humidified atmosphere with 5% CO_2_ at 37°C. Serum-starvation was carried out for 24 hours. Cells were subsequently treated for another 24 hours, washed in PBS and fixed with 4% paraformaldehyde for 10 minutes at room temperature. Cells were subsequently permeabilised in 0.4% Triton-X for 10 minutes and blocked in 3% bovine serum albumin (BSA), 5% goat serum in PBS for 1 hour. Following three washes with PBS, 2 minutes each, cells were incubated with appropriate antibodies for two hours. The primary antibodies were washed off in PBS and cells incubated with 1:100 dilution of secondary antibodies, FITC- or PE-conjugated (Alexa Fluor 488 and Alexa Fluor 555, Invitrogen, UK) for another two hours. Again, three washes were performed and the side walls of the chambers carefully removed. Coverslips were mounted onto each slide using three drops of mounting medium containing 4,6-Diamidino-2-phenylindole (DAPI) which is a highly sensitive nucleic acid stain. Images were captured using confocal laser scanning microscopy Zeiss 700. Secondary antibody alone was used to correct for non-specific background.

### Migration study

A549 cells were seeded in 96-well plates and allowed to form a confluent monolayer for 24 hours. Homogenous scratch wounds were introduced using a WoundMaker (Essen Bioscience, UK). The wound closure was monitored for up to 24 hours and data analysed using Incucyte Zoom Live Cell Imaging System (Essen Bioscience, UK). Two metric parameters were analysed: wound confluence, which reports the confluence of cells within the wound region, given as the percentage of the wound region area occupied by cells; and relative wound density, which relies on measuring the spatial cell density in the wound area relative to the spatial cell density outside of the wound area at a given time point.

### Statistical analysis

All data in the figures are presented as mean values ± SEM and all experiments have been repeated independently at least twice with 3-6 technical replicates. Statistical analysis was performed between two treatment groups by unpaired Student's t-test, and between multiple treatment groups by one-way analysis of variance (ANOVA) with Tukey post-hoc testing or two-way ANOVA with Bonferroni post-hoc test, using Graphpad Prism 5 software. The mean values of various parameters were considered to be significantly different when the p value was calculated to be less than 0.05.
